# Obstructive Sleep Apnea as a Modifiable Contributor to Chronic Pain in Rheumatologic Disease: The Potential Role of Continuous Positive Airway Pressure Therapy

**DOI:** 10.7759/cureus.113582

**Published:** 2026-07-29

**Authors:** Sanzana Tasneem, Werner Fernandes, Hakam Sulaiman, Khaled M Aldhuaina, Faris Fuqaha, Tito Gianni, Zoya Morani

**Affiliations:** 1 School of Medicine, New Vision University, Tbilisi, GEO; 2 Department of Medicine, American University of the Caribbean School of Medicine, Cupecoy, SXM; 3 Department of Internal Medicine, Faculty of Medicine, Kuwait University, Kuwait City, KWT; 4 Department of Medicine, Dammam Medical Complex, Dammam, SAU; 5 Department of Nephrology, Thomas Jefferson University Hospital, Philadelphia, USA; 6 Department of Medicine, Washington University of Health and Science, San Pedro, BLZ

**Keywords:** central sensitization, chronic pain, continuous positive airway pressure, obstructive sleep apnea, rheumatologic disease, sleep fragmentation, systemic inflammation

## Abstract

Obstructive sleep apnea (OSA) is a common sleep-related breathing disorder marked by recurrent episodes of upper-airway obstruction, leading to intermittent hypoxia, recurrent arousals, and fragmented sleep. It is highly prevalent worldwide and remains underdiagnosed, particularly when symptoms such as fatigue, poor concentration, and nonrestorative sleep overlap with obesity, chronic pain, depression, or inflammatory disease. These diagnostic challenges are clinically relevant in rheumatology, where pain, fatigue, poor sleep, and immune activation frequently coexist. This narrative review examines OSA as a potentially modifiable contributor to chronic pain and fatigue in rheumatologic disease. We synthesize evidence on intermittent hypoxia, oxidative stress, systemic inflammation, cytokine-mediated nociceptor sensitization, impaired endogenous pain inhibition, and central sensitization. Disease-specific evidence is reviewed for rheumatoid arthritis, psoriatic arthritis, systemic lupus erythematosus, axial spondyloarthritis, and fibromyalgia. We also evaluate continuous positive airway pressure (CPAP) therapy, distinguishing established benefits for respiratory events, oxygenation, sleepiness, and sleep-related quality of life from less certain pain-related effects. Current evidence suggests that CPAP may improve inflammatory biomarkers, fatigue, and pain sensitivity in selected populations, but direct rheumatology-specific evidence for pain reduction remains limited. An evidence-informed clinical framework is proposed to guide targeted OSA screening, diagnostic referral, treatment as adjunctive care, and follow-up in patients with persistent pain, fatigue, or nonrestorative sleep despite otherwise having appropriate rheumatologic management. As much of the available evidence is observational or mechanistic, future prospective studies should include standardized OSA testing, rheumatology-relevant pain outcomes, inflammatory biomarkers, disease activity measures, and long-term CPAP adherence.

## Introduction and background

Obstructive sleep apnea (OSA) is a common sleep-related breathing disorder characterized by recurrent upper-airway narrowing or collapse during sleep, resulting in intermittent hypoxia, recurrent arousals, and fragmented sleep [1,2]. Common predisposing factors include obesity, older age, male sex, craniofacial abnormalities, macroglossia or upper-airway narrowing, hypothyroidism, acromegaly, and tonsillar hypertrophy, all of which may increase the risk of upper-airway collapse during sleep [1,2]. OSA affects a substantial proportion of adults worldwide. Literature-based estimates suggest that approximately 936 million adults aged 30-69 years have mild-to-severe OSA, while approximately 425 million have moderate-to-severe disease [3].

Despite its high prevalence, OSA remains underdiagnosed in multiple patient populations. This underrecognition may reflect nonspecific symptoms such as fatigue, poor concentration, morning headache, insomnia, and nonrestorative sleep; overlap with obesity, depression, chronic pain, and inflammatory disease; limited access to polysomnography or home sleep apnea testing; and low clinical suspicion in patients who do not present with classic excessive daytime sleepiness [1,2]. These factors are particularly relevant in rheumatology clinics, where fatigue, pain, poor sleep, and mood symptoms are often attributed to the underlying rheumatologic disease itself.

Recent research has increasingly examined the relationship between sleep disturbance and chronic pain. In OSA, intermittent hypoxemia, sleep fragmentation, oxidative stress, inflammatory cytokine activity, autonomic dysregulation, and altered nociceptive processing may contribute to increased pain sensitivity [4,5]. These mechanisms may lower pain thresholds, weaken endogenous pain inhibition, and exacerbate peripheral and central sensitization [4,5]. Central sensitization refers to increased responsiveness of central nervous system pain pathways, in which pain becomes amplified despite limited peripheral tissue injury or controlled peripheral inflammation. This concept is clinically important in rheumatology because some patients report persistent pain and fatigue even when objective inflammatory activity appears controlled [5,6].

OSA is clinically important because it is identifiable and treatable. Continuous positive airway pressure (CPAP) is the standard therapy for moderate-to-severe OSA and improves airflow during sleep, reduces nocturnal hypoxemia, and can improve sleep quality [1,2]. Some studies also show reductions in inflammatory markers such as tumor necrosis factor-alpha (TNF-α) and CRP with CPAP use [7-9]. However, whether these biological changes translate into clinically meaningful pain improvements remains uncertain. In patients with persistent pain, fatigue, or poor sleep despite appropriate disease control, identifying OSA may help explain part of the remaining symptom burden.

The novelty of this review lies in integrating sleep medicine, pain neurobiology, and rheumatology into a single clinical framework. Rather than examining OSA, inflammation, chronic pain, or CPAP therapy in isolation, this review focuses on OSA as a potentially modifiable symptom amplifier in rheumatologic disease and proposes a targeted screening and follow-up approach for patients with persistent pain or fatigue despite otherwise appropriate disease management.

Objectives

This narrative review examines OSA as a possible contributor to chronic pain and fatigue in rheumatologic disease. It discusses shared biological mechanisms, disease-specific evidence, and the possible role of CPAP therapy in improving sleep, inflammation, fatigue, function, and pain-related outcomes. It also proposes an evidence-informed clinical framework for targeted OSA screening and follow-up in selected rheumatology patients.

## Review

Methodology

This article was conducted as a focused narrative review rather than a systematic review. PubMed, Google Scholar, and the Cochrane Library were searched for English-language publications available up to May 2026. Search terms included combinations of “obstructive sleep apnea”, “sleep-disordered breathing”, “chronic pain”, “fatigue”, “central sensitization”, “fibromyalgia”, “psoriatic arthritis”, “rheumatoid arthritis”, “systemic lupus erythematosus”, “spondyloarthritis”, and “continuous positive airway pressure”. Relevant review articles, observational studies, clinical trials, mechanistic studies, systematic reviews, and meta-analyses were considered if they addressed OSA, chronic pain, fatigue, inflammatory pathways, rheumatologic disease, or CPAP therapy. Reference lists of relevant articles were also manually screened to identify additional sources.

Findings were organized thematically according to mechanisms, disease-specific evidence, CPAP-related outcomes, clinical screening, and limitations relevant to rheumatology practice. As this was a narrative review, no formal systematic review protocol, independent duplicate screening, standardized data extraction form, meta-analysis, or risk-of-bias assessment was performed. The retrieved evidence was narratively synthesized to develop an evidence-informed framework describing how OSA may contribute to pain and fatigue in selected rheumatologic populations. The proposed framework is intended as a clinical approach based on available literature and has not been prospectively validated. Institutional review board approval was not required because this article used only previously published literature and did not involve patient-level data.

Mechanisms linking OSA to chronic pain

Intermittent Hypoxia, Oxidative Stress, and Systemic Inflammation

OSA is characterized by recurrent episodes of upper airway obstruction during sleep, resulting in repetitive cycles of hypoxia and reoxygenation, commonly referred to as intermittent hypoxia [1,2]. These hypoxia-reoxygenation cycles can promote oxidative stress, endothelial dysfunction, sympathetic nervous system activation, and systemic inflammatory signaling [4,5]. Intermittent hypoxia can activate nuclear factor kappa B (NF-κB), a transcription factor that regulates inflammatory gene expression and promotes production of pro-inflammatory mediators such as IL-6, TNF-α, and CRP [10]. Quantitative synthesis has also reported an association between OSA and elevated CRP levels, further supporting a link between OSA and systemic inflammation [9].

These inflammatory and oxidative processes are clinically relevant because they may extend beyond cardiometabolic complications and influence pain pathways. Sleep disruption and immune activation are closely connected, and disturbed sleep can affect inflammatory activity across physiologic systems [11]. Increased exhaled nitric oxide has also been reported in patients with OSA, suggesting that airway inflammation may occur alongside systemic inflammation [12]. Recurrent oxygen desaturation, oxidative stress, and immune activation may therefore create a biological environment in which pain pathways become more susceptible to sensitization.

Cytokine-Mediated Peripheral Nociceptor Sensitization

Repeated airway obstruction in OSA may increase the release of inflammatory mediators that influence pain signaling. Pro-inflammatory cytokines, including IL-1β, IL-6, and TNF-α, can affect neuronal activity in the superficial dorsal horn and disturb the balance between excitatory and inhibitory signaling [13]. These changes may lower pain thresholds and contribute to hyperalgesia and allodynia [13].

A similar cytokine-driven process is relevant in rheumatologic disease. In rheumatoid arthritis (RA), inflammatory cytokines contribute to joint pain, systemic symptoms, and physical limitations. However, pain intensity does not always correspond closely with the degree of active joint inflammation [14,15]. In patients with established inflammatory pain pathways, coexisting OSA may provide an additional source of inflammatory and nociceptive sensitization. This may help explain why some patients with inflammatory arthritis report pain that appears greater than expected from measured disease activity alone.

Sleep Fragmentation and Impaired Descending Pain Inhibition

OSA disrupts sleep through recurrent arousals, intermittent oxygen desaturation, and reduced restorative sleep. Slow-wave sleep helps regulate immune activity, neuroendocrine function, and pain control [5]. When sleep is repeatedly interrupted, endogenous pain-inhibitory pathways may function less effectively. This may increase pain sensitivity even when there is no new injury or clear worsening of inflammatory arthritis [5].

A systematic review and meta-analysis found that poor sleep is associated with impaired endogenous pain modulation [16]. This supports the concept that sleep disruption can reduce normal pain-inhibitory control and increase pain sensitivity. Data from rheumatology populations support this pattern. Patients with RA and psoriatic arthritis (PsA) who report severe pain frequently also report poor sleep, fibromyalgia, depression, and sleep apnea [15]. Therefore, sleep fragmentation in OSA may worsen chronic pain through two overlapping pathways: increasing systemic inflammatory signaling and disrupting central pain-inhibitory control.

Experimental sleep-deprivation studies provide additional support for a sleep-pain mechanism, although they should not be interpreted as direct OSA-specific evidence. A meta-analysis in healthy participants found that sleep deprivation increased pain sensitivity even in the absence of an underlying inflammatory disease [17]. Staffe et al. demonstrated that total sleep deprivation impaired conditioned pain modulation, facilitated temporal summation of pain, and increased sensitivity to cold and pressure stimuli [18]. Haack et al. also reported that experimental sleep disturbance altered pain-related pathways, including cyclooxygenase and endocannabinoid pathways [19]. Overall, these findings support the view that sleep fragmentation may amplify pain sensitivity through impaired descending inhibition and altered central pain processing. In OSA, these mechanisms may operate alongside intermittent hypoxia and systemic inflammation.

Central Sensitization and Nociplastic Pain

Beyond peripheral sensitization and impaired descending inhibition, chronic sleep disturbance may also contribute to central sensitization. As described earlier, central sensitization refers to increased responsiveness of central nervous system pain pathways, where pain becomes amplified despite minimal peripheral tissue pathology or controlled peripheral inflammation. This mechanism is relevant to nociplastic pain, in which altered pain processing contributes to persistent symptoms that may be disproportionate to objective inflammatory or structural findings.

Altered neurotransmitter systems may contribute to central pain amplification. Dysfunction in glutamatergic, serotonergic, dopaminergic, and inhibitory signaling pathways has been described in fibromyalgia and may contribute to widespread pain, fatigue, nonrestorative sleep, and altered stress processing [20]. Nonrestorative sleep is also a central feature of fibromyalgia, further supporting the connection between sleep disruption and centrally mediated hyperalgesia [6]. More broadly, sleep deficiency has been linked to chronic pain, and sleep disturbance has been associated with impaired pain modulatory function [5,16].

This central mechanism is particularly relevant in rheumatology, where patients may experience generalized pain even when objective inflammatory activity is minimal or controlled. In such cases, untreated OSA may worsen nociplastic pain through repeated arousals, poor restorative sleep, and altered central pain processing. Therefore, OSA should be considered as a potential amplifier of centrally mediated pain phenotypes rather than only as a respiratory comorbidity.

Interaction With Rheumatologic Inflammation

The mechanisms described above are especially relevant in rheumatologic disease because inflammation, fatigue, poor sleep, mood symptoms, and altered pain processing frequently coexist. Autoimmune and inflammatory disorders may share overlapping pathways with OSA, including immune activation, metabolic dysfunction, and systemic inflammation [21]. Inflammatory arthritis and related rheumatologic conditions involve pre-existing cytokine-mediated pain signaling, while OSA may add intermittent hypoxia, oxidative stress, sleep fragmentation, and impaired pain modulation to the inflammatory burden already present.

This interaction may help explain why some patients report persistent pain and fatigue despite treatment for the underlying rheumatologic disease. In patients with RA and PsA, intense pain has been linked to poor sleep, fibromyalgia, depression, and sleep apnea [15]. This suggests that symptom burden may reflect overlapping contributors rather than inflammatory disease activity alone. This is clinically important because patient-reported pain, fatigue, and global assessment scores may influence measured disease activity and treatment decisions.

**Figure 1:**
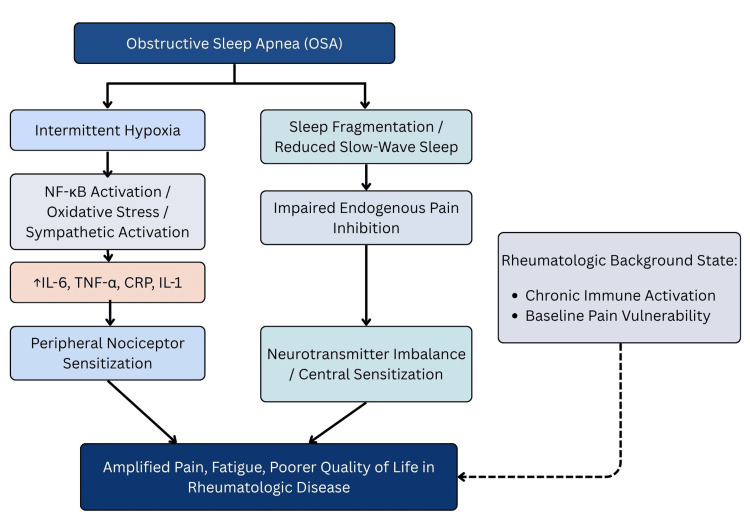
Pathophysiological model linking OSA to pain amplification. This figure was created by the authors using Canva (Canva Pty Ltd., Sydney, Australia) and was developed based on mechanisms described in the cited literature [1,2,4,5,10-13,16,20,21]. It summarizes how intermittent hypoxia and sleep fragmentation in OSA may contribute to oxidative stress, systemic inflammatory activation, impaired pain inhibition, and central sensitization. These processes may interact with underlying rheumatologic disease, potentially contributing to increased pain, fatigue, and reduced quality of life. NF-κB, nuclear factor kappa B; OSA, obstructive sleep apnea; TNF-α, tumor necrosis factor-alpha

In some patients, untreated OSA may add to the existing pain, fatigue, and poor sleep. This does not mean that OSA directly causes autoimmune disease or replaces standard assessment of inflammation. Instead, OSA may interact with existing immune and pain pathways, making symptoms more difficult to interpret. The mechanisms discussed above offer possible explanations for why patients with OSA may experience higher levels of pain sensitivity and symptom burden. These pathways are summarized in Table 1 and Figure 1.

**Table 1:** Pathophysiologic mechanisms linking OSA to pain amplification in rheumatologic disease. GABA, gamma-aminobutyric acid; NF-κB, nuclear factor kappa B; OSA, obstructive sleep apnea; TNF-α, tumor necrosis factor-alpha

Underlying mechanism	OSA-related factor	Key mediators/physiological effects	Effect of pain processing
Intermittent hypoxia and systemic inflammation	Recurrent upper airway obstruction with hypoxia-reoxygenation [1,2]	NF-κB activation, oxidative stress, sympathetic activation, and increased IL-6, TNF-α, and CRP [4,5,9-12]	Inflammation-driven pain sensitization [4,5,13]
Inflammatory-driven peripheral sensitization	Cytokine release during intermittent hypoxia [10,13]	IL-1β, IL-6, and TNF-α lower nociceptor thresholds and enhance pain transmission [13]	Hyperalgesia and persistent pain sensitization [13-15]
Sleep fragmentation and impaired pain inhibition	Recurrent micro-arousals and loss of slow-wave sleep [1,2,5]	Disrupted sleep continuity, impaired pain modulation, and reduced descending inhibition [5,15-19]	Increased pain sensitivity and fatigue [5,15-19]
Neurotransmitter imbalance and central sensitization	Chronic sleep disturbances and persistent nociceptive input [5,6,16,20]	Altered GABA, glutamate, serotonin, and dopamine signaling; neuronal hyperexcitability [20]	Central sensitization; increased pain perception [5,6,16,20]
Overlap with rheumatologic disease processes	Sleep-disordered breathing superimposed on chronic immune activation [15,21]	Shared cytokine pathways in OSA and autoimmune/inflammatory disease [15,21]	Amplifies baseline inflammation and pain [15,21]

Disease-specific evidence of OSA in rheumatologic disease

Shared Neuroimmune and Sleep-Related Pathways

Patients with rheumatologic disease often report pain, fatigue, poor sleep, and low mood together. These symptoms are not always explained by active inflammation alone. They may also reflect joint damage, medication effects, metabolic disease, altered pain processing, mood symptoms, or a separate sleep disorder. OSA may add to this symptom cluster by fragmenting sleep, increasing autonomic activity, promoting systemic inflammation, and reducing pain tolerance. Poor sleep has been linked to chronic pain through changes in pain modulation, which helps explain why nonrestorative sleep can worsen pain even when underlying inflammation has not increased [5,16]. At the same time, sleep disruption may trigger immune activation and inflammatory signaling [11]. For this reason, the relevance of OSA in rheumatology extends beyond cardiometabolic risk and may influence patient-reported pain, fatigue, and daily function.

OSA should not be viewed as a direct marker of autoimmune inflammation. Instead, it should be considered a treatable comorbidity that may amplify symptoms, particularly when patient-reported pain or fatigue appears disproportionate to objective measures of inflammatory disease activity. The relationship between OSA and autoimmune disease is not uniform across all rheumatologic conditions [21]. Therefore, pain, fatigue, and functional limitation should be interpreted within the clinical context of the specific disease rather than treated as one single disease-independent pattern.

RA: Residual Pain, Fatigue, and OSA

RA is a useful example because pain and fatigue may persist even when joint inflammation appears controlled. In a polysomnography-based study, Wali et al. reported a high prevalence of OSA among patients with RA, suggesting that sleep-disordered breathing may be missed in this group [22]. Shen et al. also found that patients with RA had a higher future risk of OSA after diagnosis [23]. Although these studies are observational, they support an association between RA and OSA beyond isolated case reports.

The main clinical issue is symptom interpretation. RA treatment decisions often rely partly on patient-reported pain, fatigue, and global assessment. These scores may remain high even when swollen joint counts and inflammatory markers improve. A register-based study linking intense pain in inflammatory arthritis to poor sleep, fibromyalgia, depression, and sleep apnea reinforces the idea that residual discomfort may reflect overlapping contributors rather than active joint inflammation alone [15].

Longitudinal data from Song et al. support the importance of sleep disturbance in RA interpretation. They followed 221 patients with active RA for 12 weeks after starting disease-modifying therapy and found that baseline sleep disturbance predicted subsequent pain intensity. They also demonstrated that central sensitization, measured using pressure pain thresholds and temporal summation, accounted for 10-19% of this relationship [24]. This effect remained significant after controlling for joint inflammatory disease activity, suggesting that sleep-related pain sensitization may operate through a pathway that is partly independent of inflammatory disease activity [24].

However, this study evaluated sleep disturbance rather than objectively diagnosed OSA. Therefore, these findings should be interpreted as support for a sleep-pain sensitization pathway rather than direct longitudinal evidence that OSA worsens RA pain. In patients with RA who continue to report persistent fatigue, nonrestorative sleep, heavy snoring, or daytime sleepiness despite controlled inflammatory disease, targeted OSA screening may help distinguish inflammatory flares from sleep-related symptom amplification.

PsA: Symptom Burden, Metabolic Risk, and Sleep Disturbance

Psoriatic disease has a close relationship with OSA because sleep disturbance, pain, fatigue, skin symptoms, obesity, metabolic comorbidity, mood symptoms, and inflammatory activity frequently overlap. A systematic review found that psoriasis is associated with several sleep disorders, including disturbed sleep quality and sleep-disordered breathing [25]. Data from a Danish cohort and a large hospital-based study also reported an association between psoriasis and OSA, including evidence that the relationship was not explained only by obesity or metabolic syndrome [26,27].

In PsA, sleep problems are common and are associated with pain, fatigue, physical function, mood symptoms, and disease-related outcomes [28]. Polysomnography-based data also support objective assessment for sleep-disordered breathing in selected PsA patients [29]. Clinically, persistent symptoms in PsA may reflect several overlapping problems, including enthesitis, skin discomfort or pruritus, obesity, depression, poor sleep, and possible OSA. Recognizing OSA may help avoid attributing all residual symptoms to uncontrolled inflammatory arthritis.

Systemic Lupus Erythematosus (SLE): Overlapping Fatigue and Sleep Pathology

SLE is a multisystem autoimmune disease in which fatigue is a major and often difficult-to-interpret symptom. Fatigue in SLE may reflect inflammatory activity, anemia, renal disease, medication effects, mood symptoms, chronic pain, reduced physical activity, or sleep disorders. A study directly examining SLE and OSA identified sleep-disordered breathing as a clinically relevant but potentially underrecognized comorbidity [30].

Recent sleep profiling in SLE supports the importance of sleep disturbance in physical and psychological outcomes. A cross-sectional study of patients with SLE identified different sleep disturbance profiles and linked them with physical and psychological outcomes [31]. These findings suggest that poor sleep may substantially worsen quality of life in patients with SLE [31]. However, a Mendelian randomization study did not find genetic evidence that sleep traits cause SLE [32]. Therefore, sleep problems may worsen daily functioning and quality of life in SLE, but they do not prove that poor sleep drives the underlying autoimmune disease process [32].

In clinical practice, the main issue is often the diagnostic overlap rather than joint pain. Fatigue may remain severe even when inflammatory markers or organ-specific lupus activity appears well controlled. Steroid-related weight gain, insomnia, sleep fragmentation, and chronic pain may also obscure coexisting OSA. Sleep apnea evaluation may be reasonable in SLE patients who have excessive daytime tiredness, loud snoring, morning headaches, witnessed apneas, or resistant hypertension. Thus, in SLE, OSA should be considered primarily as a potential contributor to fatigue, nonrestorative sleep, and quality-of-life impairment rather than as evidence of autoimmune disease activity.

Axial Spondyloarthritis: Nocturnal Pain and Conflicting OSA Evidence

The relationship between axial spondyloarthritis, including ankylosing spondylitis, and OSA requires careful interpretation. Sleep is already disrupted in these patients because of inflammatory back pain, nocturnal stiffness, and restricted mobility. Poor sleep in ankylosing spondylitis, therefore, should not automatically be attributed to OSA.

Several studies have reported a possible association between ankylosing spondylitis and OSA. Some cohort and clinical studies link OSA risk to reduced chest expansion, neck circumference, and occiput-to-wall distance [33,34]. However, Wiginder et al. did not find a higher prevalence of OSA in ankylosing spondylitis compared with the general population and reported that overlap was largely explained by age and BMI [35]. Walsh et al. also reported a lower frequency of OSA among spondyloarthritis patients receiving TNF-inhibitor therapy, although the study design limits how strongly this can be interpreted [36].

These inconsistent findings may reflect differences in study design, OSA definitions, diagnostic methods, sample size, age distribution, BMI, disease severity, and treatment exposure. In some patients, axial skeletal restriction, reduced chest expansion, or cervical posture may plausibly contribute to sleep-disordered breathing, whereas in others, OSA risk may be driven primarily by traditional factors such as older age and obesity. Treatment status may also influence observed associations, as inflammation control and TNF-inhibitor exposure could modify sleep quality or respiratory symptoms. Therefore, the relationship between axial spondyloarthritis and OSA should be interpreted as heterogeneous rather than uniformly causal [33-36].

The practical point is that OSA may coexist with axial disease, but inflammatory back pain and stiffness remain major sleep disruptors. Screening is most appropriate when typical OSA symptoms are present, such as snoring, witnessed apneas, choking or gasping during sleep, morning headaches, or persistent daytime sleepiness despite improved control of axial symptoms.

Fibromyalgia and Central Sensitization: The Sleep-Pain Phenotype

Fibromyalgia is best understood as a central sensitization or nociplastic pain phenotype rather than as localized joint inflammation. It is characterized by amplified central pain processing, widespread pain, fatigue, cognitive symptoms, and nonrestorative sleep. Poor sleep can worsen pain vulnerability and reduce endogenous pain modulation [5,16]. Fibromyalgia can also coexist with RA, PsA, SLE, or axial spondyloarthritis, making symptom interpretation more difficult when objective inflammation is low.

The relationship between fibromyalgia and OSA is clinically important because both conditions may involve fatigue, nonrestorative sleep, impaired daily function, and elevated pain sensitivity. A systematic review and meta-analysis reported clinical overlap between fibromyalgia and OSA-hypopnea syndrome [37]. Additional observational and sleep-clinic studies suggest that OSA is relatively common among patients with fibromyalgia [38-40].

Although sleep apnea does not directly cause fibromyalgia, untreated OSA may mimic or worsen fibromyalgia-like symptoms, including severe fatigue, waking unrefreshed, morning headaches, and widespread pain. In rheumatology clinics, untreated OSA may be mistaken for medication failure, uncontrolled inflammatory disease, or primary fibromyalgia. Screening for OSA is reasonable when pain appears disproportionate to objective measures of inflammatory disease activity, especially if typical symptoms such as morning headaches, loud snoring, witnessed apneas, or excessive daytime sleepiness are present.

CPAP as a modifiable intervention

Mechanism of CPAP

For patients with moderate-to-severe OSA, CPAP remains a standard treatment. The device delivers pressurized air through a mask to prevent upper-airway collapse during sleep. By reducing obstructive respiratory events, CPAP improves oxygenation and reduces sleep fragmentation. These effects are clinically important because intermittent hypoxia and disrupted sleep have both been linked to systemic inflammation and altered nociceptive processing.

The established benefits of CPAP are strongest for reducing obstructive respiratory events, improving oxygenation, reducing daytime sleepiness, and improving sleep-related quality of life in appropriately selected patients with OSA [8]. In contrast, the role of CPAP as a pain-modifying intervention remains less certain. Improvements in fatigue, inflammatory markers, and pain sensitivity provide biologically plausible pathways, but these effects should be interpreted as indirect or hypothesis-generating in rheumatologic populations unless supported by disease-specific interventional trials.

CPAP and Inflammatory Pathways

CPAP therapy may help reverse some inflammatory changes seen in OSA, including elevated cytokine levels and oxidative stress. A meta-analysis by Xie et al. demonstrated that CPAP significantly reduced circulating inflammatory markers in patients with OSA, and subsequent meta-analyses of randomized trials have reported similar reductions in CRP, IL-6, and TNF-α with sustained treatment [7,8]. These changes are clinically relevant, because lower inflammatory activity may reduce nociceptor sensitization and possibly decrease pain-related symptom burden.

However, findings from other OSA treatments should be interpreted separately from CPAP evidence. For example, Hedberg et al. reported that oral appliance therapy reduced the apnea-hypopnea index in OSA, although its impact on inflammatory biomarkers appeared limited [41]. Therefore, oral-appliance findings support the broader relevance of treating sleep-disordered breathing but should not be used as direct evidence for CPAP-mediated anti-inflammatory or pain-related effects.

The anti-inflammatory effects of CPAP also appear to depend on sustained and consistent use [8]. Therefore, short-term or irregular CPAP use is unlikely to produce durable changes in inflammatory signaling or pain modulation.

CPAP and Pain-Related Outcomes

Most CPAP research focuses on sleepiness, cardiometabolic outcomes, quality of life, and inflammatory markers rather than pain as a primary outcome. While direct rheumatology-specific evidence remains limited, studies from related populations provide mechanistic signals that treating OSA may influence fatigue, symptom burden, and pain sensitivity.

For example, Khadadah et al. reported that CPAP significantly reduced fatigue and daytime sleepiness in patients with multiple sclerosis and concomitant sleep apnea [42]. This is indirect evidence from a neurologic population rather than a rheumatologic population. Fatigue and daytime sleepiness are not direct pain outcomes, but they may influence function, symptom tolerance, and coping with pain in daily life.

A mechanistic study by Khalid et al. examined pain sensitivity in severe OSA [43]. Patients with severe untreated OSA had lower pressure pain thresholds than healthy control subjects, improved after starting CPAP therapy, and worsened after CPAP withdrawal. This pattern suggests that untreated OSA may contribute to reversible changes in pain sensitivity. However, these findings should be interpreted cautiously because the study was small and did not include patients with rheumatologic disease. Therefore, Khalid et al. support a biologically plausible CPAP-responsive pain sensitivity pathway but do not establish that CPAP directly reduces chronic pain in rheumatologic populations [43].

More targeted clinical trials are needed to determine whether CPAP leads to clinically meaningful improvements in pain outcomes in patients with rheumatologic disease.

CPAP Adherence

For patients with OSA to benefit from CPAP, adherence to therapy and appropriate patient selection are essential. Despite advances in device design, comfort, and behavioral support programs, CPAP adherence remains challenging. Approximately one third of patients do not achieve adequate adherence, and recent data suggest that adherence rates have not meaningfully improved over time [44].

Chronic pain may further complicate CPAP adherence. Jaoude et al. demonstrated that 12-month CPAP adherence was significantly lower in veterans diagnosed with OSA and comorbid chronic pain than in those without chronic pain. Adherence was 37% in patients with chronic pain compared with 55% in those without chronic pain, and greater pain intensity was the only independent predictor of non-adherence [45]. This suggests that patients with the highest symptom burden may also be among the least likely to maintain long-term CPAP use.

Rheumatology patients may face additional practical challenges that are not always captured in general CPAP adherence studies. Hand pain or joint deformity may make mask adjustment difficult, sicca symptoms may worsen discomfort from airflow-related dryness, skin sensitivity may affect mask tolerance, and persistent fatigue may reduce motivation to adhere to a new nightly treatment routine. These issues should be presented as clinically plausible barriers rather than proven rheumatology-specific adherence predictors, because direct evidence in rheumatologic populations remains limited. 

Patient Selection and Practical Limitations

Due to these limitations, patients with OSA should not be expected to experience pain improvement with CPAP in all cases. CPAP should be used as a supporting treatment and not a replacement for standard disease-modifying therapy or assessment of inflammatory activity. Patients who may benefit most from OSA-directed evaluation are those with persistent fatigue, nonrestorative sleep, or high pain scores despite well-controlled inflammatory disease and appropriate rheumatologic treatment [15,46]. In contrast, patients whose pain is clearly driven by active inflammatory disease are unlikely to experience meaningful pain relief from CPAP in isolation, even if they also have OSA.

The evidence base for CPAP as a pain-modifying intervention has several important limitations. A systematic review by McCarthy et al. evaluated positive airway pressure therapy for chronic pain in patients with OSA and found that only 10 studies met inclusion criteria from nearly 2000 screened records [47]. The authors concluded that heterogeneity in the available evidence limited the ability to determine whether PAP therapy consistently improves chronic pain or pain-related outcomes [47].

This highlights a major mismatch between sleep-medicine outcomes and rheumatology-relevant outcomes. Most CPAP trials focus on respiratory indices, cardiometabolic function, daytime sleepiness, quality of life, or inflammatory biomarkers. These are important health outcomes, but they do not directly address how rheumatologists assess pain, disease activity, fatigue, function, patient global assessment, analgesic use, or the need for escalation of anti-inflammatory treatment. This mismatch supports the need for studies using pain severity, fatigue, patient global assessment, disease activity scores, inflammatory markers, and long-term CPAP adherence as predefined endpoints.

The anti-inflammatory and symptomatic benefits of CPAP also require consistent, long-term use rather than occasional or irregular use. Zhu et al. found that decreases in circulating inflammatory marker levels were associated with sustained CPAP use [8]. In addition, educational, behavioral, troubleshooting, and telemonitoring-based interventions can improve adherence to PAP therapy among individuals diagnosed with OSA [48]. Similar adherence-enhancing strategies should be implemented proactively at the time of diagnosis rather than only after treatment difficulties arise.

**Figure 2:**
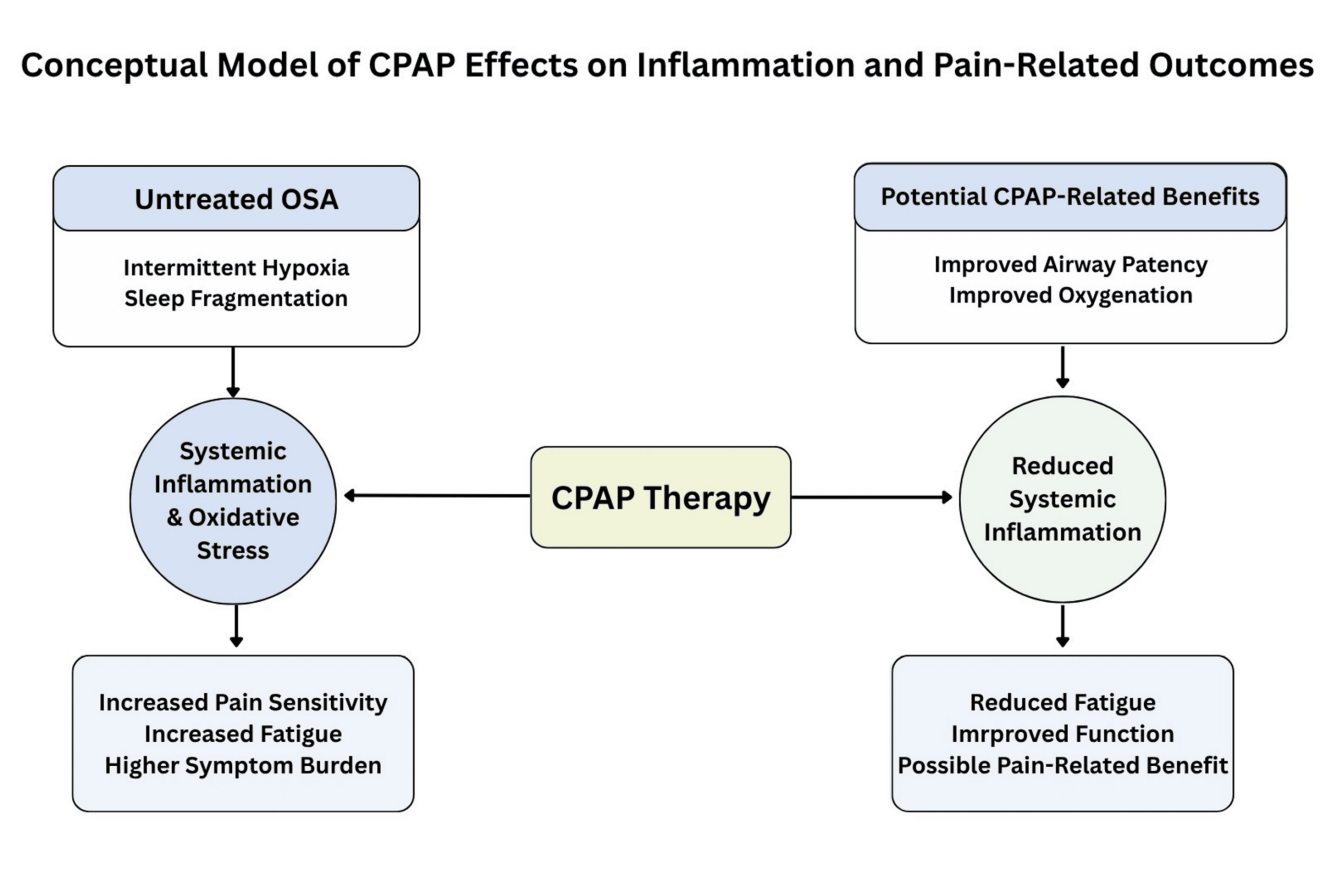
Conceptual model of CPAP effects on inflammation and pain-related outcomes. This figure was created by the authors using Canva (Canva Pty Ltd., Sydney, Australia) and was developed based on mechanisms and treatment effects described in the cited literature [7,8,41-43,46-48]. It illustrates how CPAP therapy may improve airway patency and oxygenation, reduce systemic inflammation, and contribute to improved fatigue, function, and possible pain-related benefits. Direct evidence for pain-specific outcomes in rheumatologic populations remains limited. CPAP, continuous positive airway pressure; OSA, obstructive sleep apnea

Altogether, the possible clinical and mechanistic impacts of CPAP on inflammation, fatigue, function, adherence, and pain-related outcomes are outlined in Table 2 and illustrated in Figure 2.

**Table 2:** Potential therapeutic effects of CPAP on pain-related outcomes. CPAP, continuous positive airway pressure; OSA, obstructive sleep apnea; QoL, quality of life; TNF-α, tumor necrosis factor-alpha

CPAP domains	Mechanism	Key effects	Relevance to pain
Airway stabilization	Prevents upper-airway collapse and reduces apnea/hypopnea burden [46]	Improved oxygenation and more stable sleep architecture [46]	Targets hypoxia and sleep fragmentation [1,2,46]
Anti-inflammatory effect	Reduction in circulating CRP, IL-6, and TNF-α with sustained treatment [7,8]	Lower systemic inflammatory signaling [7,8]	Decreased nociceptor sensitization and inflammation [7,8,41]
Sleep restoration	Less fragmentation and improved sleep continuity [5,8,16]	Improved daytime sleepiness, fatigue, and QoL domains [5,8,16]	Improves endogenous pain inhibition and reduces fatigue-related symptoms [5,16]
Functional outcomes	Improved daytime symptoms, fatigue, and pain sensitivity reported in selected populations [8,42,43]	Better physical function and symptom tolerance [8,42,43]	May improve physical function and pain-related QoL domains but indirect evidence [8,42,43]
Limitation of evidence	Few pain-specific trials; small samples; most studies focus on cardiometabolic endpoints [47]	Evidence remains suggestive rather than definitive [47]	Evidence remains indirect in rheumatologic populations [47]

Clinical screening and management framework for rheumatology practice 

The following framework is proposed as an evidence-informed clinical approach rather than a validated rheumatology-specific screening algorithm. Its purpose is to help identify selected patients in whom OSA may contribute to persistent pain, fatigue, or sleep disturbance despite otherwise appropriate rheumatologic care.

Identifying Rheumatology Patients Who May Benefit From OSA Screening

Routine screening for OSA in asymptomatic adults is not currently recommended. Instead, the U.S. Preventive Services Task Force supports the use of clinical judgment when deciding which patients should undergo evaluation [49]. In rheumatology practice, this supports a targeted approach, particularly in patients with persistent fatigue, nonrestorative sleep, or symptoms that appear disproportionate to objective measures of inflammatory disease activity. Pain and fatigue are often attributed solely to the underlying rheumatologic condition, which may delay recognition of coexisting sleep-disordered breathing [15].

Evidence from RA, PsA, and SLE supports this selective approach. In newly diagnosed, untreated RA and PsA, sleep-related breathing disorders and excessive daytime sleepiness have been identified using the Epworth Sleepiness Scale and cardiorespiratory polygraphy, suggesting that clinically relevant sleep-disordered breathing may be present early in inflammatory arthritis [50]. Registry data in RA and PsA also link poor sleep, fibromyalgia, depression, and sleep apnea with higher patient-reported pain and fatigue, including in patients with low objective disease activity [15]. In PsA, sleep problems are common and associated with pain, fatigue, physical function, and psychological burden [28]. In SLE, objective sleep studies suggest that OSA and poor sleep quality may contribute to fatigue in a disease where symptoms are often multifactorial [30].

In clinical practice, OSA screening should be considered in patients with loud snoring, witnessed apneas, excessive daytime sleepiness, morning headaches, obesity, resistant hypertension, nonrestorative sleep, or persistent fatigue [49]. Additional rheumatology-specific triggers include high pain scores or elevated patient global assessments despite low swollen joint counts, controlled inflammatory markers, or apparently adequate disease-modifying therapy, particularly when fibromyalgia-like pain features or depressive symptoms coexist [15,28]. In these settings, OSA may represent a modifiable contributor to persistent symptom burden rather than a reason to escalate immunosuppression alone.

Screening and Diagnostic Pathway

In rheumatology clinics, brief screening questionnaires can help identify patients who warrant formal evaluation for OSA, but they should not be treated as diagnostic tests. Snoring, Tiredness, Observed Apnea, high blood Pressure, Body mass index, Age, Neck circumference, and Gender (STOP-Bang) is a practical eight-item tool incorporating snoring, tiredness, observed apneas, blood pressure, BMI, age, neck circumference, and sex. A systematic review and meta-analysis of 47 studies involving 26,547 participants found that a STOP-Bang score of at least three had high sensitivity for detecting OSA, with sensitivities of 92% for all OSA, 95% for moderate-to-severe OSA, and 97% for severe OSA [51]. Its specificity is lower, supporting its use as a triage tool rather than a diagnostic test [51].

The Epworth Sleepiness Scale may be used to assess daytime sleepiness. However, referral decisions should not depend on sleepiness alone because some patients with OSA primarily report fatigue, nonrestorative sleep, or cardiometabolic risk factors rather than excessive daytime drowsiness [52].

A definitive diagnosis requires objective sleep testing. Home sleep apnea testing may be appropriate for uncomplicated adults with symptoms suggestive of moderate-to-severe OSA [52]. If home testing is negative, inconclusive, or technically inadequate despite ongoing suspicion, in-laboratory polysomnography should be considered. Polysomnography is also preferred when comorbid conditions may reduce the accuracy of home testing [52]. Repeat testing may be appropriate when symptoms continue despite treatment, when there is substantial weight change, or when treatment response remains uncertain [53].

Management, Monitoring, and Practical Barriers

After OSA has been diagnosed, treatment should complement rather than replace rheumatologic management. PAP therapy is recommended for adults with OSA and excessive daytime sleepiness and may also be considered when OSA affects sleep-related quality of life [54]. Early follow-up is important because education, troubleshooting, behavioral support, and telemonitoring strategies can improve adherence [48].

Practical issues should be discussed early because they can affect long-term adherence. Common problems include mask discomfort, air leaks, nasal or oral dryness, difficulty obtaining equipment, financial obstacles, and challenges maintaining regular nightly use [55-57]. As discussed above, rheumatology patients may face additional practical barriers, including hand pain or deformity affecting mask adjustment, sicca symptoms or airflow-related dryness, skin irritation, and fatigue that reduces motivation to maintain regular nightly treatment. These issues should be addressed through mask refitting, humidification, patient education, troubleshooting, and early follow-up.

Monitoring should include both sleep-related and rheumatology-relevant outcomes. Clinicians should assess CPAP/PAP adherence, residual daytime sleepiness, nonrestorative sleep, fatigue, pain severity, patient global assessment, functional status, inflammatory markers, and whether symptoms continue to appear disproportionate to objective disease activity. CPAP should be framed as adjunctive care for confirmed OSA rather than as a replacement for disease-modifying therapy or standard rheumatologic assessment.

The proposed approach to screening, referral, treatment, and follow-up is summarized in Table 3.

**Table 3:** Evidence-informed clinical framework for OSA screening and follow-up in rheumatologic patients with persistent pain or fatigue. CPAP, continuous positive airway pressure; DMARDs, disease-modifying antirheumatic drugs; ESR, erythrocyte sedimentation rate; OSA, obstructive sleep apnea; PAP, positive airway pressure; QoL, quality of life; STOP-Bang, Snoring, Tiredness, Observed Apnea, high blood Pressure, Body mass index, Age, Neck circumference, and Gender

Framework step	When to consider it	Recommended action
(1) Assess rheumatologic disease activity	Persistent pain, fatigue, poor sleep, or high patient global assessment [15,28]	Review joint examination, disease activity score, CRP/ESR, medication response, and comorbid pain contributors [15,28]
(2) Identify an OSA risk phenotype	Snoring, witnessed apneas, daytime sleepiness, morning headaches, obesity, resistant hypertension, nonrestorative sleep, or fatigue disproportionate to disease activity [49]	Consider targeted OSA screening rather than routine testing of all rheumatology patients [49]
(3) Apply screening tools	OSA symptoms or risk factors are present [51,52]	Use STOP-Bang and/or Epworth Sleepiness Scale as triage tools, not diagnostic tests [51,52]
(4) Confirm OSA objectively	Positive screening or high clinical suspicion [52,53]	Refer for home sleep apnea testing or polysomnography according to sleep medicine guidance [52,53]
(5) Treat confirmed OSA as adjunctive care	Objective diagnosis of OSA [54]	Use CPAP/PAP alongside standard rheumatologic therapy, not as a replacement for DMARDs or anti-inflammatory treatment [54]
(6) Monitor response and barriers	After CPAP/PAP initiation or if symptoms persist [48,55-57]	Track adherence, residual sleepiness, fatigue, pain, QoL, disease activity, inflammatory markers, and mask-related barriers [48,55-57]

Limitations of the available evidence and future directions

Limitations of the Available Evidence

Several limitations should be considered when interpreting the evidence summarized in this review. First, much of the literature linking OSA with pain and rheumatologic symptom burden is observational, which limits causal interpretation. Associations between OSA, fatigue, poor sleep, fibromyalgia-like symptoms, and pain did not prove that OSA directly worsens rheumatologic disease activity or that treating OSA will reliably reduce pain.

Second, the available studies are heterogeneous in terms of rheumatologic diagnosis, OSA definitions, diagnostic methods, pain measures, inflammatory markers, and follow-up duration. Some studies rely on questionnaires or administrative data, whereas others use polysomnography or cardiorespiratory polygraphy. This variability makes it difficult to compare findings across RA, PsA, SLE, axial spondyloarthritis, and fibromyalgia.

Third, CPAP studies rarely use pain severity, patient global assessment, rheumatologic disease activity scores, or analgesic and immunosuppressive treatment changes as primary outcomes. Most CPAP trials focus on respiratory indices, sleepiness, cardiometabolic outcomes, quality of life, or inflammatory biomarkers. As a result, the evidence supporting CPAP as a pain-modifying intervention in rheumatologic populations remains indirect.

Finally, adherence is a major practical limitation. Any potential anti-inflammatory or pain-modulatory effect of CPAP is likely to depend on sustained use, but long-term adherence may be difficult in patients with chronic pain, fatigue, sicca symptoms, skin sensitivity, or hand deformities affecting mask adjustment. These limitations support the need for prospective, disease-specific studies using standardized OSA testing, pain outcomes, inflammatory markers, disease activity measures, and adherence data.

Gaps in Literature and Future Directions

Existing studies suggest an association between OSA, sleep disturbance, inflammation, fatigue, and pain. However, the available evidence remains limited, and several clinically important questions remain unanswered. Most current research focuses on cardiovascular outcomes, daytime sleepiness, respiratory indices, quality of life, and inflammatory biomarkers, with fewer studies evaluating pain as a prespecified primary outcome.

In rheumatologic disease, the strongest data suggest associations between poor sleep, fatigue, fibromyalgia-like symptoms, and worse pain, especially in patients with RA and PsA [15,28]. However, these findings are mainly associative and do not prove that OSA directly worsens rheumatologic pain or disease activity. Current studies do not clearly establish whether CPAP treatment reduces pain severity, improves patient global assessment, or changes rheumatologic disease activity.

Several disease-specific questions remain unanswered. Associations between OSA and RA, PsA, SLE, axial spondyloarthritis, and fibromyalgia have been reported, but the strength and clinical significance of these relationships appear to differ between conditions. Future clinical trials should stratify participants by rheumatologic diagnosis, objective inflammatory activity, fibromyalgia or nociplastic pain features, OSA severity, BMI, and baseline fatigue burden. This approach would help clarify whether OSA treatment is most useful for patients with inflammatory pain, centrally mediated amplification, fatigue-dominant presentations, or mixed symptom phenotypes.

The evidence for CPAP as a pain-modifying intervention also remains indirect. CPAP has demonstrated benefits for sleepiness, quality of life, and inflammatory markers in OSA; however, direct evidence for pain-specific outcomes in rheumatologic populations remains limited [7,8,42,47]. Future trials should include rheumatology-relevant endpoints such as pain severity, fatigue, patient global assessment, disease activity scores, sleep quality, functional status, inflammatory biomarkers, analgesic use, immunosuppressive treatment escalation, and long-term CPAP adherence.

A clearer understanding of which patient groups benefit most would help define the role of sleep-directed interventions as adjunctive care in rheumatology practice. Future studies should clarify whether targeted OSA screening and sustained CPAP adherence improve outcomes beyond standard rheumatologic care, particularly in patients whose pain, fatigue, or sleep disturbance appears disproportionate to objective inflammatory disease activity.

## Conclusions

OSA is a common and treatable condition that may worsen chronic pain and fatigue in selected patients with rheumatologic disease through intermittent hypoxia, oxidative stress, systemic inflammation, impaired descending pain inhibition, and central sensitization. Current evidence supports OSA as a potential symptom amplifier rather than a proven direct cause of rheumatologic pain. CPAP therapy may improve sleep quality, oxygenation, daytime symptoms, inflammatory profiles, fatigue, and functional status, but direct evidence for pain reduction in rheumatologic populations remains limited. CPAP should therefore be viewed as an adjunctive intervention rather than a replacement for standard rheumatologic treatment.

Targeted OSA screening should be considered in patients with persistent fatigue, nonrestorative sleep, classic OSA symptoms, or elevated patient-reported pain despite otherwise appropriate rheumatologic management. Future prospective studies should determine whether sustained CPAP adherence improves pain, fatigue, function, inflammatory biomarkers, and other rheumatology-relevant outcomes.
